# Parental Socialization, School Adjustment and Cyber-Aggression among Adolescents

**DOI:** 10.3390/ijerph16204005

**Published:** 2019-10-19

**Authors:** Belén Martínez-Ferrer, Celeste León-Moreno, Daniel Musitu-Ferrer, Ana Romero-Abrio, Juan Evaristo Callejas-Jerónimo, Gonzalo Musitu-Ochoa

**Affiliations:** Department of Education and Social Psychology, Pablo Olavide University, 41013 Seville, Spain; bmarfer2@upo.es (B.M.-F.); dmusfer@alu.upo.es (D.M.-F.); aromabr@upo.es (A.R.-A.); jecaljer@upo.es (J.E.C.-J.); gmusoch@upo.es (G.M.-O.)

**Keywords:** parental socialization, school adjustment, cyber-aggression, adolescence

## Abstract

The objective of the present study is to analyse the relationships between parental socialization styles—indulgent, authoritarian, authoritative and negligent, school adjustment (social integration, academic competence and family involvement) and cyber-aggression (direct and indirect) in adolescents. Participating in this study were 1304 Spanish students of both sexes (53.1% girls), aged between 12 and 18 years (*M* = 13.87, *SD* = 1.33). Multivariate analyses of variance were performed. The results showed significant relationships between parental socialization styles, school adjustment and cyber-aggression. It was observed that adolescents from indulgent and authoritative families showed greater academic competence and greater family involvement. Additionally, the children from authoritarian families displayed greater involvement in direct and indirect cyber-aggression behaviours. The results obtained and their implications are discussed in the final section.

## 1. Introduction

Socialization is defined as a learning and internalization process through which the values, habits and cultural norms necessary for adaptation to a given society are acquired [1,2,3,4]. One of the most relevant socialization contexts, especially in childhood and adolescence, is family [5,6,7,8]. In the field of family socialization, parental styles are among the constructs to have received most attention among researchers. Since the first studies conducted by Baumrind [9,10], a two-dimensional model has been consolidated, based on two orthogonal axes: responsiveness or involvement/acceptance, and demandingness or severity/imposition [11,12,13]. Based on the combination of both axes, four parental styles have been identified [12,13,14,15,16]: indulgent (high involvement/acceptance and low severity/imposition); authoritative (high degree of involvement/acceptance and high severity/imposition); authoritarian (low involvement/acceptance and high severity/imposition); and negligent (low involvement/acceptance and low severity/imposition). Authoritative and indulgent styles have been found to be associated with better adjustment in children in different spheres, although cultural differences exist regarding the effectiveness of these styles [17]. While in Anglo-Saxon culture the authoritative style has been identified as the most associated with the adjustment of children [10,11,13,16,18,19], in Mediterranean culture the indulgent style is the most adaptive [14,20,21,22].

In relation to adjustment at school, it has been observed that parents have a significant influence on the academic and social success of students [23,24]. School adjustment is defined as the ability of students to adapt to the educational context and includes aspects such as academic performance, adaptation to school standards, respect for teachers as figures of authority, student attitude towards school and participation in school activities [25,26,27]. According to Cava, Povedano, Buelga, and Musitu [28], school adjustment in adolescence is associated with collaboration and involvement on the part of families in the educational processes and experiences of their children [29,30]. Families in schools transmit positive values and attitudes to their children towards education and teachers [31] which, in turn, is associated with the greater academic effort of children, greater participation in school tasks and more positive relationships with peers and teachers [32,33,34].

Previous studies have reported that parents who use an indulgent or authoritative style enhance the development of positive attitudes towards the school environment and education in their children, aspects on which the latter's school adjustment is built [35,36]. This relationship seems to be due to fact that both parental styles are based on support and affection [37]. Moreover, both styles are characterized by high responsiveness and affection, which are expressed through greater frequency of compliment and praise related to children's academic performance, greater dialogue and positive and empathic communication. These practices have a positive impact on the development of adolescents' academic, social and behavioural skills, thus contributing to better school adjustment [6,38,39,40,41,42,43,44,45,46]. Most studies carried out in Anglo-Saxon cultural contexts confirm the relevance of the authoritative style in promoting the school adjustment of children [13,15,47,48]. However, the indulgent style seems to have a more positive effect on school adjustment in European and Latin American countries such as Spain [49], Portugal [3], Italy [50], Germany [51], Brazil [51] or Mexico [52].

Another important variable considered in this study, due to its relationships with both parental styles and school adjustment [53,54,55,56,57], is cyber-aggression, which is defined as aggressive and intentional behaviour through the use of digital media [58,59,60]. Cyber-aggression can be direct (e.g., sending harmful messages and/or images to the victim without the need for the aggressor to hide his/her identity) or indirect (e.g., the use of third parties to carry out actions or send masked messages) [61,62]. Cyber-aggression might have a negative influence on the behaviors and quality of life of adolescents who spend a lot of time on the Internet [63]. Although studies analysing the relationship between parental styles and cyber-aggression are scarce [64], it has been observed that the use of inadequate patterns of family communication and coercion are associated with greater cyber-aggression [61,64,65,66], while positive family relationships, characterized by affection and inductive, positive, open and empathic communication reduce the likelihood of children becoming engaging in cyber-aggressive behaviours [57,67,68]. One limitation of these studies is that cyber-aggression is analysed globally. However, the analysis of direct and indirect cyber-aggression could help explain the different causes and associated correlates, to the extent that both types of behaviour differ in terms of the degree of planning and involvement of other individuals [69].

Regarding gender differences, it has been observed that girls display a more positive attitude towards schoolwork and spend more time doing these tasks than boys [70,71,72]. In addition, girls are more oriented towards the fulfilment of tasks and goals [73,74], which has important repercussions on their academic results [75,76,77]. Girls have also been observed to build more positive interpersonal relationships in the classroom, which is associated with better school adjustment [78,79]. As regards cyber-aggression, available empirical evidence offers inconclusive results. It has been observed that boys tend to be more violent on the Internet than girls [80,81,82,83,84,85,86]. However, other studies have reported no significant differences according to gender [87,88]. Another important aspect that enables a more detailed analysis of gender differences are the different types of cyber-aggression. 

Accordingly, the general objective of the present study was to analyse the relationships between parental socialization styles (indulgent, authoritarian, authoritative and negligent), school adjustment (social integration, academic competence and family involvement) and cyber-aggression (direct and indirect) in adolescents. The following hypotheses were considered:

**H1**: *Indulgent and authoritative styles are associated with greater school adjustment (social integration, academic competence and family involvement) than authoritarian and negligent styles*.

**H2**: *The authoritarian style is related to greater direct and indirect cyber-aggression, compared to the indulgent, authoritative and negligent styles*.

**H3**: *Indulgent and authoritative styles will result in greater school adjustment and less cyber-aggression in girls than in boys*.

## 2. Materials and Methods 

### 2.1. Participants and Procedure

The sample consisted of 1304 adolescents of both sexes (53.1% girls) aged between 12 and 18 (*M* = 13.87, *SD* = 1.33), enrolled at four Compulsory Secondary Education (ESO) centres in the autonomous communities of Andalusia, Aragon and Valencia (Spain). For the selection of the sample, random group sampling was used in the geographical area of Andalusia, Aragon and the Autonomous Community of Valencia. The primary units of the sample were the urban and rural areas in the three autonomous communities. The secondary units were public and private secondary schools in each area. Classes were not treated as tertiary units, as all classes from the first to the fourth year at the selected centres were included in the study. A series of prior analyses of differences in means yielded the variables the object of the study, as a function of the location of the centres and their public or private status. No statistically significant differences were observed. 

Data for this research were compiled as part of a broader study on violent behaviour in adolescents in Spain (Reference: PSI2015-65683-P). The study was approved by the ethical committee of each participating university (DPS.EEL.01.15). Additionally, the study complies with the ethical values required in research with human beings and respects the fundamental principles included in the Helsinki Declaration. Once permission had been obtained from the educational centres to carry out the research and active informed consent from the families had been granted, the battery of instruments was administered in two different sessions of approximately 45 minutes at the educational centres.

### 2.2. Instruments

Parenting Styles. The Parental Socialization Scale (ESPA29, [14]) was used based on the two-dimensional theoretical model of parental socialization [12,15]. This scale consists of 232 items that measure, on a response scale ranging from 1 (never) to 4 (always), parents' performance in 29 situations that are representative of everyday family life in Western culture: 13 that represent situations of obedience with family norms (e.g., “If I take care of my things and am clean and properly dressed”) in which adolescents value the frequency with which parents show affection (e.g., “He/she shows warmth”), α = 0.96; indifference (e.g., “He/she seems indifferent”), α = 0.95; and 16 that refer to situations of disobedience with these norms (e.g., “If I leave home to go somewhere without asking anyone for permission”) in which adolescents rated the frequency with which parents respond through dialogue (e.g., “He/she talks to me”), α = 0.95; indifference (e.g., “It's the same to him/her”), α = 0.90; verbal scolding (e.g., “He/she scolds me”), α = 0.94; physical punishment (e.g., “He/she hits me”), α = 0.94; and revoking privileges (e.g., “He/she takes something away from me”), α = 0.95. The family score in acceptance/involvement was obtained by averaging the responses for affection, dialogue, indifference and dissatisfaction (in the last two sub-scales the responses were inverted by being negatively related with the dimension). The family severity/imposition score was obtained by averaging responses in verbal scolding, physical punishment and revoking privileges [13,15]. Both family indices ranged between 1 and 4 points, high levels of acceptance/involvement and severity/imposition corresponding to high scores. Based on these scores, the family socialization style was defined as authoritative, indulgent, authoritarian or negligent. Cronbach's alpha in acceptance/involvement was α = 0.90, and the values obtained in severity/imposition were α = 0.96.

School Adjustment. The Scale of Teacher's Perception of School Adjustment (PROF-A, [28]) was used. This scale consists of 13 items that measure teachers' perception of student adjustment based on a response scale ranging from 0 (very low/very bad) to 9 (very high/very good). The scale consists of three dimensions: social integration (e.g., “The student's relationship with his/her classmates”), α = 0.92; academic competition (e.g., “The student's interest in and attention to what is done in class”), α = 0.95; and family involvement (e.g., “The degree of the family's involvement in the school monitoring of the child”), α = 0.93. Cronbach's alpha for the scale was α = 0.93. 

Cyber-aggression. The Cyber-Aggression Scale (CybAG_R, [58]) was used. This scale consists of 24 items that measure involvement in violent behaviours through the use of digital media during the last 12 months, based on a response scale ranging from 1 (never) to 5 (many times/more than 10 times). The scale consists of two dimensions: direct cyber-aggression (e.g., “I have taken a person's mobile phone and used it to send embarrassing photos, videos or messages to get them into trouble”), α = 0.94; and indirect cyber-aggression (e.g., “I have passed myself off as someone else to do bad things on the Internet or using a mobile phone”), α = 0.86. Cronbach's alpha for the scale was α = 0.94.

### 2.3. Data Analysis

Firstly, the distribution of the families was calculated according to the educational style, as well as the means and standard deviations obtained in each of the dimensions of the model (see Table 1). Subsequently, a multivariate factorial design (MANOVA, 4 × 2) was carried out, with the SPSS statistical program (version 20, Pablo de Olavide University, Seville, Andalusia, Spain) considering parental socialization styles (indulgent, authoritative, authoritarian and negligent) and gender (boys and girls) as fixed factors to analyse the possible effects of interaction. The three dimensions of teachers' perception of students (social integration, academic competence and family involvement) and the two dimensions of the cyber-aggression scale (direct and indirect) were considered as dependent variables. Finally, univariate tests (ANOVAS) were calculated to study the statistically significant differences in the variables and the Bonferroni post-hoc test (α = 0.05) was performed.

## 3. Results

### 3.1. Multivariate Factor Analysis 

In the MANOVA, statistically significant differences were found in the main effects of parental socialization styles (Λ = 0.94, *F*(15, 3081.19) = 4.63, *p* < 0.001, η^2^*_p_* = 0.02); and gender (Λ = 0.95, *F*(5, 1116) = 12.05, *p* < 0.001, η^2^*_p_* = 0.05). Three statistically-significant interaction effects were also observed between parental socialization styles and gender (Λ = 0.97, *F*(15, 3081.19) = 2.54, *p* < 0.05, η^2^*_p_* = 0.01) (see Table 2).

### 3.2. Parental Socialization Style

The ANOVA revealed significant differences in academic competence (*F*(3, 1147) = 6.512, *p* < 0.001, η^2^*_p_* = 0.02, family involvement (*F*(3, 1300) = 7.904, *p* < 0.001 η^2^*_p_* = 0.02), direct cyber-aggression (*F*(3, 1300) = 14,312, *p* < 0.001, η^2^*_p_* = 0.03), and indirect cyber-aggression (*F*(3, 1300) = 12.709, *p* < 0.001, η^2^*_p_* = 0.03) (see Table 3). The Bonferroni tests (α = 0.05) indicated that children from indulgent and authoritative families obtained statistically higher scores in academic competence than children with authoritarian parents. Additionally, the children from indulgent and authoritative families obtained statistically higher scores in family involvement than the children with authoritarian and negligent parents. The children from authoritarian families obtained statistically higher scores in the two dimensions of cyber-aggression (direct and indirect) compared to the children from indulgent, authorizing and neglectful families. 

### 3.3. Demographic Variable: Gender

The ANOVA revealed significant differences with respect to gender in the social integration variables (*F*(1, 1126) = 14,380, *p* < 0.001, η^2^*_p_* = 0.01), academic competence (F (1,149) = 37,925, *p* < 0.001, η^2^*_p_* = 0.03) and direct cyber-aggression (*F*(1, 1302) = 11,469, *p* < 0.01, η^2^*_p_* = 0.01). As shown in Table 4, the Bonferroni tests (α = 0.05) indicated that the girls obtained higher scores in social integration and academic competence, while the boys obtained higher scores in direct cyber-aggression. 

### 3.4. Interaction Analysis

**Figure 1:**
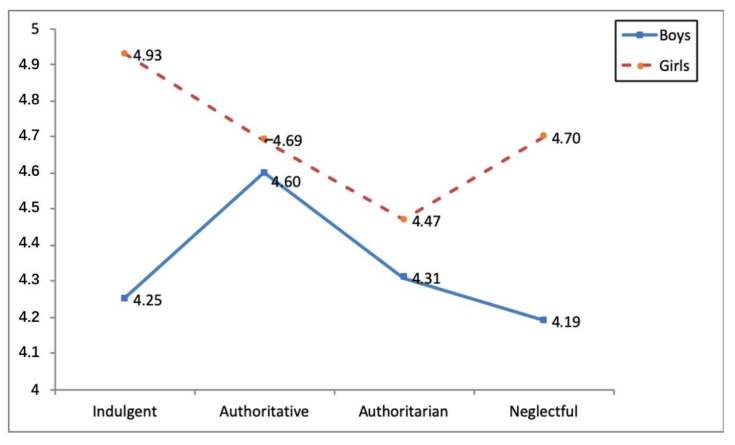
Interaction effect parenting style x gender and social integration.

**Figure 2:**
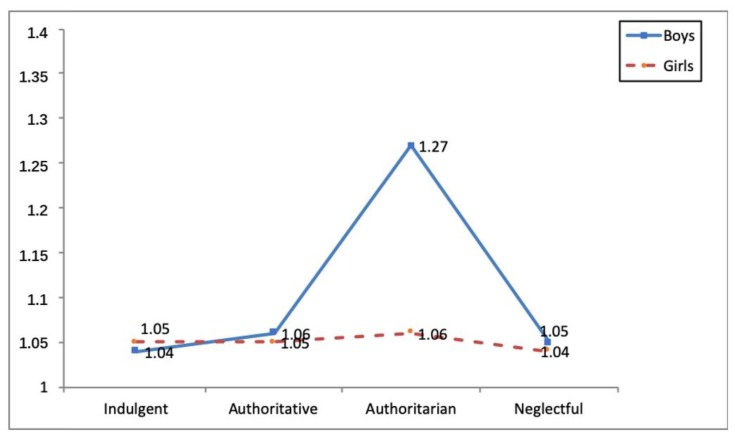
Interaction effect parenting style x gender and direct cyberaggression.

**Figure 3:**
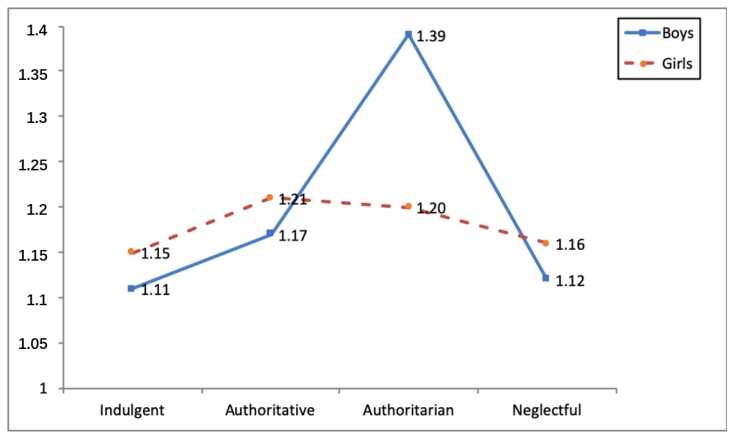
Interaction effect parenting style x gender and indirect cyberaggression.

Three statistically-significant interaction effects were identified between parental socialization styles and gender in the social integration variable (*F*(3, 1120) = 3.19, *p* < 0.05, η^2^*_p_* = 0.01), direct cyber-aggression (*F*(3, 1120) = 8.57, *p* < 0.001, η^2^*_p_* = 0.02) and indirect cyber-aggression (*F*(3, 1120) = 5.71, *p* < 0.01, η^2^*_p_* = 0.02) (see Table 5). As regards the first interaction, girls educated in indulgent families obtained higher social integration scores than boys from families employing the same parental style and those from negligent families (see Figure 1). In terms of the second and third interaction, the analyses carried out a posteriori showed that authoritatively-educated boys obtained the highest direct and indirect cyber-aggression scores (see Figure 2 and Figure 3). 

## 4. Discussion

The objective of the present study was to analyse the relationships between parental socialization styles (indulgent, authoritarian, authoritative and negligent), school adjustment (social integration, academic competence and family involvement) and cyber-aggression (direct and indirect) in adolescents. The results obtained indicated that adolescents from families using indulgent or authoritative styles displayed greater academic proficiency and family involvement. However, no significant differences in social integration were observed according to the style of socialization, thus partially confirming the first hypothesis. Previous studies have highlighted that indulgent and authoritative styles enhance teenagers' academic proficiency [89,90,91], probably due to greater parental involvement in school [92]. In this sense, the participation of families in the educational context of their children promotes the latter's success at school, more positive self-esteem and greater self-confidence, aspects that, in turn, promote school adjustment [31,93,94,95,96]. However, social integration has proven to be equivalent in all four styles, probably because parents are more involved in academic and performance-related issues and focus more on this social dimension when problems of school integration emerge. These results underscore the importance of analysing school adjustment from a multi-dimensional perspective taking into account aspects that transcend academic performance. More research is needed to analyse the role of family variables on social integration.

With respect to cyber-aggression, as hypothesized, adolescents from authoritarian families showed greater involvement in direct and indirect cyber-aggressive behaviours, while involvement in such behaviours was similar in adolescents brought up by parents employing an indulgent, authoritative and negligent style. These results are in line with the conclusions reported in previous research that the authoritarian style is the most associated with involvement in violent behaviour in adolescence [55,57,81].

The parental practices of authoritarian families are characterized as being coercive and imposed, and based on submission, obedience and control, with a low level of affection and involvement, expressed through cold and empathic communication. These findings suggest that adolescents from authoritarian families transfer this type of practice to their relationships with peers in the virtual environment [97] and consider direct and indirect forms of cyber-aggression to be acceptable [61,62,98,99].

In this sense, the results infer that the adolescents most involved in cyber-aggression behaviours often come from families in which low levels of affection and coercion prevail, probably because the virtual space constitutes an environment beyond the eyes of adults. 

The results for the third hypothesis indicated that girls from indulgent families showed greater social integration than boys from indulgent and negligent families, while boys from authoritarian families obtained the highest scores in both direct and indirect cyber-aggression; hence, the hypothesis was partially confirmed. Although girls displayed greater school adjustment in terms of social integration and academic proficiency, the interaction effect revealed that girls from indulgent families (with high levels of affection and discipline based on communication and self-revelation) were the most socially integrated at school. These results infer that both the school environment and family relationships constitute socialization scenarios in which gender has a transversal effect. Thus, for adolescent girls, social relationships, both with peers and with teachers, as well as emotional involvement in family and at school, acquire greater importance than for boys [100].

Therefore, the confluence of parental practices with formal school socialization processes seems to enhance social integration in girls, who also usually present adequate academic proficiency. Thus, positive attitudes towards school imply more positive social relationships [101,102,103], greater academic effort and greater participation in school tasks [40,73,74,94,104,105], aspects which are socially reinforced in girls. Furthermore, previous studies have shown that girls display greater empathy and pro-sociability in the educational context [106,107], aspects that seem to facilitate their social integration in the classroom. 

In contrast, boys from authoritarian families showed the highest levels of involvement in cyber-aggression (direct and indirect) compared to the other groups studied. These results are in line with those reported in previous studies [80,81,82,83,84,85,86]. In this sense, the results obtained here suggest that in the authoritarian style, based on coercion coupled with poor communication, the socialization of gender roles fosters greater assimilation of traditional patterns in which coercion and imposition are less censored traits in boys [98,99,100], resulting in aggression and hostile behaviours being perceived as legitimate or justifiable behaviours of “masculinity” [108,109]. Cyberaggression could partly explain why adolescent males tended to report that frequent Internet use affected their health [110]. The authors of this study believe that future research should incorporate gender socialization measures linked to socialization styles for a more in-depth analysis of socialization styles and their implications. 

## 5. Limitations

Finally, the results of the present study have certain limitations. The cross-sectional nature of the study design did not allow casual relationships to be established. Therefore, future research incorporating different time panels would allow us to explore the differences identified in the study. In view of the cultural differences observed with respect to socialization styles and their relationship with the adjustment of children, it would be worthwhile carrying out further research in other countries in southern Europe, in order to compare the results obtained and thus draw conclusions regarding the influence of parental socialization on school adjustment and children's involvement in cyber-aggression in the Mediterranean context. Future studies should use other statistical techniques that make it possible to control variables such as the socio-educational level and occupation of the parents. Additionally, in the present study, the importance of the gender of the teachers in the perception of the school adjustment of the children—as well as the time of Internet use, was not analysed and could be addressed in future research.

## 6. Conclusions

The results of this research show that parental socialization styles play a key role in the school and behavioural adjustment of adolescents. The findings show that parenting styles defined as high acceptation/involvement, such as the indulgent and authoritative styles, are related to better school adjustment and low involvement in both direct and indirect cyberaggression. Thus, affection, parental involvement, empathic communication and dialogue enhance the school adjustment of children, probably because parents who embrace such socialization styles are also involved in the schoolwork and social relations of their children, thus promoting the development of academic, social and behavioural skills that are valued positively at school. 

Therefore, results of the present study have important implications for practice. Evidence from this study highlights that family–school interactions in the individual, group and institutional spheres should be promoted. Positive and fluid communication between parents and teachers could contribute positively to students, not only at academic level but also in relation to such relevant issues as behavioural problems in the classroom and on the Internet. Finally, the findings of this study confirm the importance of considering the influence of gender socialization processes, which are transmitted to boys and girls, in the design of educational programs and strategies to prevent cyber-aggression.

## Figures and Tables

**Table 1:** Numbers of cases in parenting style groups, as well as mean scores and standard deviations on measures of parental dimensions.

	Total Sample	Indulgent	Authoritative	Authoritarian	Neglectful
Frequency	1304	367	323	275	339
Percent	100	28	25	21	26
Acceptance/Involvement					
Mean	3.13	3.39	3.49	2.80	2.78
SD	0.43	0.26	0.25	0.30	0.32
Severity/Imposition					
Mean	1.98	1.77	2.31	2.27	1.64
SD	0.38	0.23	0.26	0.27	0.23
Sociodemographic variables					
Occupation Yes	923	231	241	195	256
Occupation No	381	136	82	80	83
Primary education	236	63	57	62	54
Secondary education	691	179	177	144	191
University	377	125	89	69	94

**Table 2:** MANOVA results for all the studied variables (4 ^a^ × 2 ^b^).

	Λ	*F*	*gl_between_*	*gl_error_*	η^2^*_p_*
(A) Parenting Style ^a^	0.94	4.63 ***	15	3081.19	0.02
(B) Gender ^b^	0.95	12.05 ***	5	1116.00	0.05
A × B	0.97	2.54 *	15	3081.19	0.01

Note: ^a^ indulgent, authoritative, authoritarian and neglectful; ^b^ boy and girl; * *p* < 0.05, *** *p* < 0.001.

**Table 3:** Means (Standard deviations), *F* values, and Bonferroni post hoc test for the parenting style groups across adolescent.

Parenting Style						
	Indulgent	Authoritative	Authoritarian	Neglectful	*F*	η^2^*_p_*
SI	4.61 (1.76)	4.65 (1.60)	4.39 (1.64)	4.47 (1.65)	1.439	0.00
AC	5.69 (2.00) ^a^	5.47 (2.00) ^a^	4.95 (2.09) ^b^	5.32 (2.02)	6.512 ***	0.02
FI	6.06 (1.71) ^a^	6.00 (1.83) ^a^	5.54 (2.13) ^b^	5.48 (2.14) ^b^	7.904 ***	0.02
DC	1.05 (0.16) ^b^	1.06 (0.18) ^b^	1.16 (0.46) ^a^	1.05 (0.16) ^b^	14.312 ***	0.03
IC	1.13 (0.26) ^b^	1.19 (0.37) ^b^	1.30 (0.51) ^a^	1.14 (0.31) ^b^	12.709 ***	0.03

Note: SI = Social Integration; AC = Academic Competence; FI = Family Involvement; DC = Direct Cyber-aggression; IC = Indirect Cyber-aggression; *** *p* < 0.001; a > b.

**Table 4:** Means (Standard deviations), *F* values, and Bonferroni post hoc test for the parenting style groups across adolescent.

Gender				
	Boys	Girls	*F*	η^2^*_p_*
SI	4.33 (1.80)	4.71 (1.51)	14.380 ***	0.01
AC	4.98 (2.04)	5.71 (1.98)	37.925 ***	0.03
FI	5.69 (2.13)	5.87 (1.80)	2.505	0.00
DC	1.10 (0.33)	1.06 (0.18)	11.469 **	0.01
IC	1.19 (0.39)	1.18 (0.35)	0.386	0.00

Note: SI = Social Integration; AC = Academic Competence; FI = Family Involvement; DC = Direct Cyber-aggression; IC = Indirect Cyber-aggression; ** *p* < 0.01, *** *p* < 0.001.

**Table 5:** Means (Standard deviations), *F* values, and Bonferroni post hoc test for the parenting style groups across adolescent.

Parenting Style							
	Gender	Indulgent	Authoritative	Authoritarian	Neglectful	*F*(3, 1120)	η^2^*_p_*
SI	Boys	4.25 (1.97) ^b^	4.60 (1.75)	4.31 (1.71)	4.19 (1.75) ^b^	3.19 *	0.01
	Girls	4.93 (1.48) ^a^	4.69 (1.47)	4.47 (1.56)	4.70 (1.53)		
DC	Boys	1.04 (0.09) ^b^	1.06 (0.20) ^b^	1.27 (0.60) ^a^	1.05 (0.13) ^b^	8.57 ***	0.02
	Girls	1.05 (0.20) ^b^	1.05 (0.16) ^b^	1.06 (0.18) ^b^	1.04 (0.19) ^b^		
DI	Boys	1.11 (0.19) ^b^	1.17 (0.36) ^b^	1.39 (0.63) ^a^	1.12 (0.21) ^b^	5.71 **	0.02
	Girls	1.15 (0.31) ^b^	1.21 (0.38) ^b^	1.20 (0.33) ^b^	1.16 (0.37) ^b^		

Note: SI = Social Integration; DC = Direct Cyber-aggression; IC = Indirect Cyber-aggression; * *p* < 0.05, ** *p* < 0.01, *** *p* < 0.001; a > b.

## References

[1] Aymerich M., Musitu-Ochoa G., Palmero F. (2018). Family Socialisation Styles and Hostility in the Adolescent Population. Sustainability.

[2] Baumrind D. (1966). Effects of Authoritative Parental Control on Child Behavior. Child Dev..

[3] Rodrigues Y., Veiga F., Fuentes M.C., García F. (2013). Parenting and Adolescents’ Self-Esteem: The Portuguese Context. J. Psychodidact..

[4] Whiting J.W. (1970). Socialization. Anthropological Aspects. Enciclopedia de las Ciencias Sociales.

[5] Martínez I., Garcia F., Fuentes C., Veiga F., Garcia O.F., Rodrigues Y., Cruise E., Serra E. (2019). Researching Parental Socialization Styles across Three Cultural Contexts: Scale ESPA29 Bi-Dimensional Validity in Spain, Portugal, and Brazil. Int. J. Environ. Res. Public Health.

[6] León-Moreno C., Musitu-Ferrer D. (2019). Family Communication Patterns, School and Family Self-Concept, and Motivation of Revenge among Adolescents. Eur. J. Investig. Health Psychol. Educ..

[7] Suárez-Relinque C., del Moral Arroyo G., León-Moreno C., Callejas Jerónimo J.E. (2019). Child-To-Parent Violence: Which Parenting Style Is More Protective? A Study with Spanish Adolescents. Int. J. Environ. Res. Public Health.

[8] Perasso G., Carone N., Lavina B., Health Behaviour in School Aged Children Lombardy Group 2014 (2019). Alcohol Consumption in Adolescence: The Role of Adolescents ’ Gender, Parental Control, and Family Dinners Attendance in an Italian HBSC Sample. J. Fam. Stud..

[9] Baumrind D. (1967). Ghild Care Practices Anteceding Three Patterns of Preschool Behavior. Genet. Psychol. Monogr..

[10] Baumrind D. (1971). Developmental Psychology Monograph Current Patterns of Parental Authority. Dev. Psychol..

[11] Darling N., Steinberg L. (1993). Parenting Style as Context: An Integrative Model. Psychol. Bull..

[12] Maccoby E.E., Martin J.A., Mussen P.H. (1993). Socialization in the Context of the Family: Parent- Child Interaction. Handbook of Child Psychology.

[13] Lamborn S.D., Mounts N.S., Steinberg L., Dornbusch S.M. (1991). Patterns of Competence and Adjustment among Adolescents from Authoritative, Authoritarian, Indulgent, and Neglectful Families. Child Dev..

[14] Musitu G., García J.F., Musitu G., García F. (2001). ESPA29: Parental Socialization Scale in Adolescence.

[15] Steinberg L., Lamborn S., Darling N., Mounts N., Dornbusch S. (1994). Over-Time Changes in Adjustment and Competence among Adolescents from Authoritative, Authoritarian, Indulgent, and Neglectful Families. Child Dev..

[16] Steinberg L., Mounts N., Lamborn S., Dornbusch S. (1991). Authoritative Parenting and Adolescent Adjustment across Various Ecological Niches. J. Res. Adolesc..

[17] Garcia F., Serra E., Garcia O.F., Martinez I., Cruise E. (2019). A Third Emerging Stage for the Current Digital Society? Optimal Parenting Styles in Spain, the United States, Germany, and Brazil. Int. J. Environ. Res. Public Health.

[18] Chao R.K. (2001). Extending Research on the Consequences of Parenting Style for Chinese Americans and European Americans. Child Dev..

[19] Dornbusch S.M., Ritter P.L., Leiderman P.H., Roberts D.F., Fraleigh M.J. (1987). The Relation of Parenting Style to Adolescent School Performance. Child Dev..

[20] García F., Gracia E. (2010). What Is the Optimum Parental Socialisation Style in Spain? A Study with Children and Adolescents Aged 10–14 Years. J. Study Educ. Dev..

[21] Martínez I., Camino L., Camino C., Cruise E., Selin H. (2014). Family Socialization in Brazil. Parenting Across Cultures. Science Across Cultures: The History of Non-Western Science.

[22] Martinez I., Garcia J.F. (2007). Impact of Parenting Styles on Adolescent Self-Esteem and Internalization. Span. J. Psychol..

[23] Benner A.D., Boyle A.E., Sadler S. (2016). Parental Involvement and Adolescents’ Educational Success: The Roles of Prior Achievement and Socioeconomic Status. J. Youth Adolesc..

[24] Brajša-Žganec A., Merkaš M., Šakić Velić M. (2019). The Relations of Parental Supervision, Parental School Involvement, and Child’s Social Competence with School Achievement in Primary School. Psychol. Sch..

[25] Ladd G.W., Burgess K.B. (2001). Do Relational Risks and Protective Factors Moderate the Linkages between Childhood Aggression and Early Psychological and School Adjustment?. Child Dev..

[26] Musitu-Ferrer D., Esteban-Ibañez M., León-Moreno C., García O.F. (2019). Is School Adjustment Related to Environmental Empathy and Connectedness to Nature?. Psychosoc. Interv..

[27] Rodríguez-Gutiérrez E., Martín-Quintana J.C., Cruz-Sosa M. (2016). “Living Adolescence in Family” Parenting Program: Adaptation and Implementation in Social and School Contexts. Psychosoc. Interv..

[28] Cava M.J., Povedano A., Buelga S., Musitu-Ochoa G. (2015). Psychometric Analysis of the Scale of Teacher’s Perception of School Adjustment (PROF-A). Psychosoc. Interv..

[29] Jeynes W.H. (2005). A Meta-Analysis of the Relation of Parental Involvement to Urban Elementary School Student Academic Achievement. Urban Educ..

[30] Sheldon S.B. (2003). Linking School-Family-Community Partnership in Urban Elementary Schools to Student Achievement on State Tests. Urban Rev..

[31] Gutiérrez M., Tomás J.-M., Romero I., Barrica J. (2017). Perceived Social Support, School Engagement and Satisfaction With School. J. Psychodidact..

[32] Dettmers S., Yotyodying S., Jonkmann K. (2019). Antecedents and Outcomes of Parental Homework Involvement: How Do Family-School Partnerships Affect Parental Homework Involvement and Student Outcomes?. Front. Psychol..

[33] Simons R.L., Simons L.G., Chen Y.F., Brody G.H., Lin K.H. (2007). Identifying the Psychological Factors That Mediate the Association between Parenting Practices and Delinquency. Criminology.

[34] Yubero S., Larrañaga E., Martínez M.I., Ovejero A., Smith P.K., Yubero S. (2013). Family, Communication and Ciberbullying. El acoso Escolar y su Prevención: Perspectivas Internacionales.

[35] Steinberg L., Blatt-Eisengart L., Cauffman E. (2006). Patterns of Competence and Adjustment Among Adolescents from Authoritative, Authoritarian, Indulgent, and Neglectful Homes: A Replication in a Sample of Serious Juvenile Offenders. J. Res. Adolesc..

[36] Pears K.C., Kim H.K., Capaldi D., Kerr D.C.R., Fisher P.A. (2013). Father-Child Transmission of School Adjustment: A Prospective Intergenerational Study. Dev. Psychol..

[37] Leijten P., Thomaes S., Orobio de Castro B., Dishion T.J., Matthys W. (2016). What Good Is Labeling What’s Good? A Field Experimental Investigation of Parental Labeled Praise and Child Compliance. Behav. Res. Ther..

[38] Crespo-Ramos S., Romero-Abrio A., Martínez-Ferrer B., Musitu-Ochoa G. (2013). Psychosocial Variables and Overt School Violence among Adolescents. Psychosoc. Interv..

[39] Pinquart M., Kauser R. (2018). Do the Associations of Parenting Styles With Behavior Problems and Academic Achievement Vary by Culture? Results From a Meta-Analysis. Cult. Divers. Ethn. Minor. Psychol..

[40] Ramos-Díaz E., Rodríguez-Fernández A., Revuelta L., Zuazagoitia A. (2016). Adolescent Students’ Perceived Social Support, Self-Concept and School Engagement. J. Psychodidact..

[41] Romero-Abrio A., León-Moreno C., Musitu-Ferrer D., Villarreal-González M.E. (2019). Family Functioning, Self-Concept and Cybervictimization: An Analysis Based on Gender. Soc. Sci..

[42] Sabey A.K., Rauer A.J., Haselschwerdt M.L., Volling B. (2018). Beyond “Lots of Hugs and Kisses”: Expressions of Parental Love From Parents and Their Young Children in Two-Parent, Financially Stable Families. Fam. Process.

[43] Totkova Z. (2019). Symbolic Interactionism and the Perceived Style of Parenting. Qual. Sociol. Rev..

[44] Tung I., Lee S.S. (2018). Context-Specific Associations Between Harsh Parenting and Peer Rejection on Child Conduct Problems at Home and School. J. Clin. Child Adolesc. Psychol..

[45] Wang C., Xia Y., Li W., Wilson S.M., Bush K., Peterson G. (2016). Parenting Behaviors, Adolescent Depressive Symptoms, and Problem Behavior: The Role of Self-Esteem and School Adjustment Difficulties Among Chinese Adolescents. J. Fam. Issues.

[46] Yeung J.W., Chen H.F., Lo H.H., Choi A.W. (2017). Relative Effects of Parenting Practices on Child Development in the Context of Family Processes. J. Psychodidact..

[47] Barber B.K., Chadwick B.A., Oeter R. (1992). Parental Behaviors and Adolescent Self-Esteem in the United States and Germany. J. Marriage Fam..

[48] Steinberg L., Elmen J.D., Mounts N.S. (1989). Authoritative Parenting, Psychosocial Maturity, and Academic Success among Adolescents. Child Dev..

[49] Moreno-Ruiz D., Estévez E., Jiménez T.I., Murgui S. (2018). Parenting Style and Reactive and Proactive Adolescent Violence: Evidence from Spain. Int. J. Environ. Res. Public Health.

[50] Di R., Carla M., Di Maggio R., Zappulla C. (2014). Mothering, Fathering, and Italian Adolescents’ Problem Behaviors and Life Satisfaction: Dimensional and Typological Approach. J. Child Fam. Stud..

[51] Wolfradt U., Hempel S., Miles J.N.V. (2003). Perceived Parenting Styles, Depersonalisation, Anxiety and Coping Behaviour in Adolescents. Personal. Individ. Differ..

[52] Villalobos J.A., Cruz A.V., Sánchez P.R. (2004). Parental Styles and Psychosocial Development in High School Students. Rev. Mex. Psicol..

[53] Bradbury S., Dubow E., Domoff S. (2018). How Do Adolescents Learn Cyber- Victimization Coping Skills? An Examination of Parent and Peer Coping Socialization. J. Youth Adolesc..

[54] Goldstein S. (2016). Adolescents’ Disclosure and Secrecy About Peer Behavior: Links with Cyber Aggression, Relational Aggression, and Overt Aggression. J. Child Fam. Stud..

[55] Gómez-Ortiz O., Apolinario C., Romera E.M., Ortega-Ruiz R. (2019). The Role of Family in Bullying and Cyberbullying Involvement: Examining a New Typology of Parental Education Management Based on Adolescents’ View of Their Parents. Soc. Sci..

[56] Hemphill S., Kotevski A., Tollit M., Smith R., Herrenkohl T., Toumbourou J., Catalano R. (2012). Longitudinal Predictors of Cyber and Traditional Bullying Perpetration in Australian Secondary School Students. J. Adolesc. Health.

[57] Martínez I., Murgui S., Garcia O.F., Garcia F. (2019). Parenting in the Digital Era: Protective and Risk Parenting Styles for Traditional Bullying and Cyberbullying Victimization. Comput. Hum. Behav..

[58] Buelga S., Ortega-Barón J., Torralba E. Psychometric Properties of the Revised Cyber-Aggression Scale Scales (CybAG-R). Proceedings of the II Congreso Internacional de la Sociedad Científica Española de Psicología Social.

[59] Del-Rey-Alamillo R., Mora-Merchán J.A., Casas J.-A., Ortega-Ruiz R., Elipe P. (2018). “Asegúrate” Program: Effects on Cyber-Aggression and Its Risk Factors. Comunicar.

[60] Álvarez García D., Barreiro Collazo A., Núñez Pérez J. (2017). Cyberaggression among Adolescents: Prevalence and Gender Differences. Comunicar.

[61] Kowalski R.M. (2008). Cyber Bullying: Recognizing and Treating Victim and Aggressor. Psychiatr. Times.

[62] Vandebosch H., Van Cleemput K. (2009). Cyberbullying among Youngsters: Profiles of Bullies and Victims. New Media Soc..

[63] Tran B.X., Huong L.T., Hinh N.D., Nguyen L.H., Le B.N., Nong V.M., Thuc V.T.M., Tho T.D., Latkin C., Zhang M.W. (2017). A Study on the Influence of Internet Addiction and Online Interpersonal Influences on Health-Related Quality of Life in Young Vietnamese. BMC Public Health.

[64] Jones L.M., Mitchell K.J., Finkelhor D. (2012). Trends in Youth Internet Victimization: Findings from Three Youth Internet Safety Surveys 2000–2010. J. Adolesc. Hetalth.

[65] Kowalski R., Morgan C., Limber S., Von Marées N., Petermann F. (2012). Traditional Bullying as a Potential Warning Sign of Cyberbullying. Sch. Psychol. Int..

[66] Lwin M.O., Li B., Ang R.P. (2012). Stop Bugging Me: An Examination of Adolescents’ Protection Behavior against Online Harassment. J. Adolesc..

[67] Appel M., Stiglbauer B., Batinic B., Holtz P. (2014). Internet Use and Verbal Aggression: The Moderating Role of Parents and Peers. Comput. Hum. Behav..

[68] Solecki S., McLaughlin K., Goldschmidt K. (2014). Promoting Positive Offline Relationships to Reduce Negative Online Experiences. J. Pediatr. Nurs..

[69] Buelga S., Martínez-Ferrer B., Cava M.-J., Ortega-Barón J. (2019). Psychometric Properties of the CYBVICS Cyber-Victimization Scale and Its Relationship with Psychosocial Variables. Soc. Sci..

[70] Deslandes R., Cloutier R. (2002). Adolescents’ Perception of Parental Involvement in Schooling. Sch. Psychol. Int..

[71] Jackson C. (2003). Motives for “Laddishness” at School: Fear of Failure and Fear of the “feminine”. Br. Educ. Res. J..

[72] Harris S., Nixon J., Rudduck J. (1993). School Work, Homework and Gender. Gend. Educ..

[73] Wagner P., Schober B., Spiel C. (2008). Time Students Spend Working at Home for School. Learn. Instr..

[74] Xu J. (2007). Middle-School Homework Management: More than Just Gender and Family Involvement. Educ. Psychol..

[75] Han F. (2019). Longitudinal Relations Between School Self-Concept and Academic Achievement. Rev. Psicodidact..

[76] Olivier E., Archambault I., Dupéré V. (2018). Boys’ and Girls’ Latent Profiles of Behavior and Social Adjustment in School: Longitudinal Links with Later Student Behavioral Engagement and Academic Achievement?. J. Sch. Psychol..

[77] Veas A., Castejón J.L., Miñano P., Gilar-Corbí R. (2019). Early Adolescents’ Attitudes and Academic Achievement: The Mediating Role of Academic Self-Concept. Rev. Psicodidact..

[78] Ewing A.R., Taylor A.R. (2018). Early Childhood Research Quarterly the Role of Child Gender and Ethnicity in Teacher—Child Relationship Quality and Children ’ s Behavioral Adjustment in Preschool. Early Child. Res. Q..

[79] Hamre B.K., Pianta R.C. (2001). Early Teacher-Child Relationships and the Trajectory of Children’s School Outcomes through Eighth Grade. Child Dev..

[80] Buelga S., Pons J. (2012). Aggressions among Adolescents through Mobile Phones and the Internet. Psychosoc. Interv..

[81] Moreno-Ruiz D., Martínez-Ferrer B., García-Bacete F. (2019). Parenting Styles, Cyberaggression, and Cybervictimization among Adolescents. Comput. Hum. Behav..

[82] Fundación ANAR (2016). Estudio Sobre Ciberbullying Según los Afectados, Informe del Teléfono.

[83] Fundación ANAR (2018). Informe del Teléfono ANAR: II Estudio Sobre Acoso Escolar y Ciberbullying Según los Afectados.

[84] Lee C., Shin N. (2017). Computers in Human Behavior Prevalence of Cyberbullying and Predictors of Cyberbullying Perpetration among Korean Adolescents. Comput. Hum. Behav..

[85] Perren S., Gutzwiller-Helfenfinger E., Malti T., Hymel S. (2012). Moral Reasoning and Emotion Attributions Ofadolescent Bullies, Victims, and Bully-Victims. Br. J. Dev. Psychol..

[86] Slonje R., Smith P.K. (2008). Cyberbullying: Another Main Type of Bullying?. Scand. J. Psychol..

[87] Katzer C., Fetchenauer D., Belschack F. (2009). Cyberbullying: Who Are the Victims? A Comparison of Victimization in Internet Chatrooms and Victimization in School. J. Media Psychol..

[88] Sentse M., Kretschmer T., Salmivalli C. (2015). The Longitudinal Interplay between Bullying, Victimization, and Social Status: Age-Related and Gender Differences. Soc. Dev..

[89] Cerezo M.T., Casanova P.F., De la Torre M.J., Carpio M.D.L.V. (2018). Parents’ Educational Styles and Self-Regulated Learning Strategies in a Group of Secondary Education Student. Eur. J. Educ. Psychol..

[90] Gracia E., Fuentes M.C., Garcia F., Lila M. (2012). Perceived Neighborhood Violence, Parenting Styles, and Developmental Outcomes among Spanish Adolescents. J. Community Psychol..

[91] Viguer P., Solé N. (2012). School and Peers as Contexts of Socialization of Values and Living Together: A Participatory Research through a Family Debate. Cult. y Educ..

[92] Cava M.J. (2011). Family, Teachers, and Peers: Keys for Supporting Victims of Bullying. Psychosoc. Interv..

[93] Sánchez-Valle M., De-Frutos-Torres B., Vázquez-Barrio T. (2017). Parent’s Influence on Acquiring Critical Internet Skills. Comunicar.

[94] Valle A., Pan I., Núñez J.C., Rosário P., Rodríguez S., Regueiro B. (2015). Homework and Academic Achievement in Primary Education. An. Psicol..

[95] Jeynes W.H. (2011). Parental Involvement and Academic Success.

[96] Martínez-Ferrer B., Musitu-Ochoa G., Murgui-Pérez S., Amador-Muñoz L.V. (2009). Marital Conflict, Family Communication, and School Adjustment in Adolescents. Rev. Mex. Psicol..

[97] Gómez P., Harris S.K., Barreiro C., Isorna M., Rial A. (2017). Profiles of Internet Use and Parental Involvement, and Rates of Online Risks and Problematic Internet Use among Spanish Adolescents. Comput. Hum. Behav..

[98] Piaget J. (1975). L’equilibration des Structures Cognitives.

[99] Bryant C., Conger R., Vangelesti A., Reis H., Fitzpatrick M. (2002). An Intergenerational Modelo f Romantic Relationship Development. Stability adn Change in Relationships.

[100] Santoro C., Martínez-ferrer B., Monreal Gimeno C., Musitu G. (2018). New Directions for Preventing Dating Violence in Adolescence: The Study of Gender Models. Front. Psychol..

[101] Cava M.J., Buelga S., Musitu G., Murgui S. (2010). School Violence between Adolescents and Their Implications in the Psychosocial Adjustment: A Longitudinal Study. J. Psychodidact..

[102] Letamendia R. (2002). Abuse in School Contexts. J. Psychodidact..

[103] Ortega-Barón J., Buelga S., Cava M., Torralba E. (2017). School Violence and Attitude Toward Authority of Students Perpetrators of Cyberbullying. J. Psychodidact..

[104] Cava M.J., Musitu G. (1999). School Integration: An Analysis of Sex Functions and the Academic Course of Students. Rev. Española Orientación y Psicopedag.

[105] Xu J., Wu H. (2013). Self-Regulation of Homework Behavior: Homework Management at the Secondary School Level. J. Educ. Res..

[106] Carlo G., Raffaelli M., Laible D.J., Meyer K.A. (2002). Why Are Girls Less Physically Aggressive than Boys? Personality and Parenting Mediators of Physical Aggression. J. Res. Adolesc..

[107] Singh-Manoux A. (2000). Culture and Gender Issues in Adolescence: Evidence from Studies on Emotion. Psicothema.

[108] Espelage D.L., Merrin G.J., Davis J.P., Rose C.A., Little T.D. (2018). A Longitudinal Examination of Homophobic Name-Calling in Middle School: Bullying, Traditional Masculinity, and Sexual Harassment as Predictors. Psychol. Violence.

[109] Plummer D. (2016). One of the Boys: Masculinity, Homophobia, and Modern Manhood.

[110] Do H., Onyango B., Prakash R., Tran B., Nguyen Q., Nguyen L., Ho R. (2019). Susceptibility and Perceptions of Excessive Internet Use Impact on Health among Vietnamese Youths. Addict. Behav..

